# Migrant Entrepreneurs as Agents of Development? Geopolitical Context and Transmobility Strategies of Colombian Migrants Returning from Venezuela

**DOI:** 10.1007/s12134-022-00959-w

**Published:** 2022-04-27

**Authors:** Yvonne Riaño

**Affiliations:** Institute of Geography and ‘nccr - on the move’, University of Neuchâtel, Neuchâtel, Switzerland

**Keywords:** Return migration, Forced Migration, Reintegration, Migrant entrepreneurship, Development, Geopolitical conflict, Coping strategies, Colombia–Venezuela border

## Abstract

Returnee entrepreneurs are often represented in migration and development discourses as agents of development. This assumes that they acquire valuable socio-economic resources abroad which help them to create successful businesses upon return. However, we have scant knowledge of the impact of the geopolitical context on returnee entrepreneurs or their coping strategies. Latin American returnees in particular have received little attention and few studies focus on migrants with ‘South-to-South’ return trajectories. Emphasising the role of territorial conflicts and the agency of individuals, I use a feminist geopolitical perspective to address these gaps. I contribute to migration, mobility, and development studies by studying whether Colombian migrants returning from Venezuela can reintegrate as successful entrepreneurs. Further, I offer the concept of transmobilities to study the cross-border nature of strategies of reintegration. The 30 returnees studied have a trajectory of repeated forced mobilities, ranging from internal displacement in Colombia, subsequent emigration to Venezuela, and final deportation to Colombia by Venezuela’s government. I combine the qualitative methods of multi-sited ethnography, biographical interviews, mental maps, and participatory *Minga* workshops. The analysis shows that Colombian returnees face intense difficulties in reintegrating despite their strong motivation and entrepreneurial spirit. The geopolitical context of armed struggle, an absent Colombian state, and territorial conflicts between Colombia and Venezuela create an unfavourable environment for returnee entrepreneurs. Consequently, they develop transmobility strategies — including the movement of people, goods, and capital across national borders — at the risk of their own lives. The simplistic discourse of returnees as agents of development needs to be revised.

## Introduction

The idea that return migrants can contribute to the development of their communities of origin has been given a powerful impulse from academics and policy-makers (King, 2022; Sørensen et al., 2002; Thomas-Hope, 1999). Returnee migrant entrepreneurship has great potential to aid reintegration and generate local employment, assuming that returning migrants of different skill levels invest their savings in local ventures that benefit from acquired international expertise (Batista et al., 2017; Marchetta, 2012; McCormick & Wahba, 2001). Returnees have thus been represented as agents of development (Åkesson, 2016; Sinatti, 2019). However, this representation seems to overlook the variation in geographical contexts to which migrants return. I argue that the geographical context — i.e. the places to which migrants return — is important because location affects the kinds of opportunity that returnee entrepreneurs can access. Addressing geopolitics in returnee contexts is particularly important because territorial conflicts are becoming increasingly acute. Most are concentrated in Asia and Africa and the most common forms are territorial disputes and civil wars (CFR, 2022). In Latin America, tensions related to Venezuela’s political regime — as well as its contiguity with Colombia’s unresolved internal conflicts — currently present major geopolitical challenges (González Levaggi, 2020). This raises the issue of how returnees exercise agency in different geopolitical contexts and how this influences their entrepreneurial projects. Thus far, we have only a limited understanding of these issues (Cassarino, 2000; Medina & Menjívar, 2015).

Not only are studies on returnee entrepreneurship scarce but reintegration through entrepreneurial ventures is not a main topic of study. Most studies examine the probability that a returnee will become an entrepreneur (e.g. Démurger & Xu, 2011; Thomas & Inkpen, 2013). Several authors conclude that returnees are more likely to become entrepreneurs than non-migrants (Batista et al., 2017; Piracha & Vadean, 2010). For McCormick and Wahba (2001), overseas savings and the duration of stay increase such a probability. Black and Castaldo (2009) conclude that the international work experience of returnees to West Africa is the most significant predictor of their entrepreneurial activity. Similarly, Marchetta (2012) states that migration experience significantly improves the prospects of survival of Egyptian returnees’ businesses. For Piracha and Vadean (2010), schooling, foreign-language proficiency, and savings from time spent abroad are the key factors conducive to entrepreneurship among Albanian returnees. This type of study is criticised for downplaying social and human indicators in favour of economic indicators; they exclude the reintegration wishes of returnees and focus mostly on surveys and statistical models (e.g. Sinatti, 2019). Furthermore, their analytical emphasis is on the economic performance of individuals rather than on framework conditions such as geopolitical contexts and few studies examine the survival strategies of returnees (Cassarino, 2000).

Due to financial crises, progressively restrictive migration policies and a lack of economic opportunities in host countries, an increasing number of Latin American migrants are returning (Maldonado Hernández et al., 2020). However, with some exceptions (Cavalcanti & Parella, 2013; Cortés & Oso, 2017; Riaño, 2013), few studies examine the return and reintegration of Latin American migrants. Furthermore, studies of migrants with ‘South-to-South’ return trajectories are particularly scarce.

This paper aims to advance our understanding of returnee entrepreneurship by examining the context-specific constraints and opportunities that Colombian migrants returning from Venezuela face to mobilise resources for sustainable entrepreneurship, as well as the coping strategies which they mobilise. The following research questions are posed:

- To what extent can returnees successfully reintegrate as entrepreneurs?
- How does the geopolitical context affect constraints and opportunities for sustainable entrepreneurship?
- What kinds of coping strategies do returnee entrepreneurs develop and how successful are they?

The empirical study focuses on returnees who have experienced repeated forced mobility, including internal displacement within Colombia, emigration to Venezuela and deportation from Venezuela to Colombia. These migrants now live on the border between Cúcuta (Colombia) and San Antonio del Táchira (Venezuela). Using multi-sited ethnography, biographic interviews, mental maps, and participatory *Minga* workshops (Riaño, 2016), I examine the reintegration situations of 30 Colombian returnees in relation to their entrepreneurship and coping strategies.

The article comprises seven sections. The first introduces the perspectives of *feminist geopolitics* and *transmobilities*, which are respectively used to examine the role of geopolitical context and the strategies of reintegration of returnees, as underpinned by evolving South–South geopolitics. The second section introduces the methodology and sites of empirical study in Colombia, while the third outlines the context of geopolitics, forced mobilities, and return. The fourth section presents the multiple types of return movements practised by the returnees and the fifth focuses on the role of entrepreneurship in their reintegration. The sixth examines the role of transmobility strategies for returnee businesses. The seventh section places the empirical findings in a wider theoretical context and poses questions for future research.

## Illuminating ‘Return Migration’ and ‘Reintegration’ from the Perspectives of Feminist Geopolitics and Transmobilities

I use a feminist geopolitical perspective (Dowler & Sharp, 2001; Massaro & Williams, 2013) to conceptualise context, thus emphasising the role of local territorial conflicts and the agency of individuals. Geopolitics is important to understanding the choices and opportunities which migrants face as they reintegrate. Moreover, feminist geopolitics questions the traditional scales of analysis of territorial struggles by ‘refocusing on the mundane, everyday reproductions of geopolitical power’ (Massaro & Williams, 2013: 567), thus highlighting everyday practices in relation to constructions of territorial borders (Dowler & Sharp, 2001). Further, it emphasises that ‘geopolitical relations are produced in the home as much as the battlefield and by a whole suite of actors outside the formal political realm’ (Massaro & Williams, 2013: 569). Rather than focusing on the state and supranational power, feminist geopolitics enables us to study ‘struggles over power, territory and security’ (2013: 572) by actors who use their agency in everyday spaces to recreate international borders. I contend that this perspective is particularly suitable for studying border zones such as that between Colombia and Venezuela, where disputes over territorial control play an important role in shaping the practices and strategies of the returnees there.

Examining the theoretical perspectives of ‘transnational migration’ (Glick Schiller et al., 1992) and ‘mobilities’ (Sheller & Urry, 2006) can be useful to advance our understanding of return movements and strategies of reintegration. Recent years have seen a lively debate on the notion of ‘return’. Researchers have criticised the simplistic conceptualisation of ‘return migration’ which depicts migrants’ move from a ‘destination country’ back to the ‘place of origin’ as the final stage of the migratory process (Ley & Kobayashi, 2005). Transnational migration moves beyond this narrow understanding by viewing returnees as socially and economically connected not only with their country of origin but also with the country of destination (King & Christou, 2014; Vathi et al., 2018), thus acknowledging ‘the increased interconnectedness between home and host countries engaged in by migrants’ (Sinatti, 2011: 154). However, return scholars have critiqued the idea that transnational interconnectedness is simply a relationship between the origin and destination countries (Cortés, 2011). As Sandoz et al., (2021: 4) contend, ‘contemporary migrants have the potential to simultaneously connect with various countries, given their multiple mobility experiences and global connections’. Cortés (2011) thus concludes that multiple and varied returns are transnational strategies undertaken by migrants and their families in order to transcend social exclusion. Moreover, the idea by some authors writing on transnational migrant entrepreneurship that returnee entrepreneurs are ‘scientists and engineers’ and that dual connectedness automatically leads to economic success (Drori et al., 2009) has been called into question. Grünhagen et al. (2020) remark that, by focusing on highly skilled migrant entrepreneurs in the technology, consulting, and educational sectors (Brzozowski et al., 2014), returnee studies have given scant attention, with some exceptions (Bolzani, 2020), to less-privileged migrants working in low-profit sectors.

The ‘mobilities’ perspective (Sheller & Urry, 2006) has the potential to overcome the limits of the transnational approach. Being a contemporary paradigm that explores the movement of people (human migration, individual mobility, travel, transport), ideas, and things, as well as the broader social implications of those movements, it makes fewer assumptions about the duration and purpose of return movements and considers all the material and immaterial flows involved in transnational spatial movements. The mobilities perspective is also advantageous as it pays specific attention to the spatial and temporal dimensions of how individuals move between different places and at different times and how they mobilise human and non-human entities across national and transnational space, an emphasis not always present in transnational studies.

**Fig. 1:**
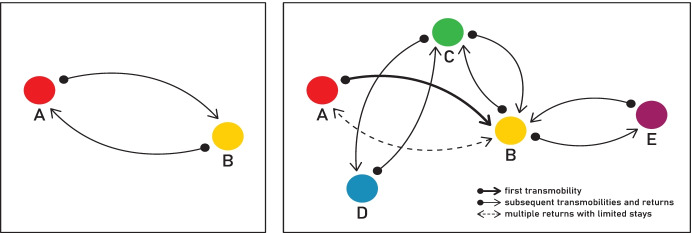
Return migration as a *closed and completed cycle* and as an *open cycle*

Bringing the ideas of transnationalism and mobilities together, I offer the concept of *transmobilities*^1^ to study return and reintegration. Transmobilities view the spatial mobility of people, goods, and capital across borders as crucial strategies facilitated by social networks, which are used by (former) migrants — in different forms and at different places and times — to improve their chances of reintegration. Thus, the perspective of transmobilities focuses not only on the spatial movements of migrants themselves but also on how they mobilise material and immaterial items across physical and virtual space. This means acknowledging that processes of re-integration are not only confined to the scale of the national state but they may also extend beyond national boundaries. Moreover, in coining the concept of transmobilities, I am particularly interested in the different kinds of spatial movement that returnees carry out between different places — thus studying how they mobilise goods, capital, and other people in physical and virtual space as a key strategy to develop their livelihoods. Scholars, such as Sinatti (2011), studying the spatial movements of returnees, have used the concept of ‘unsettled returnees’ to study the alternate mobilities of migrants in Europe and Senegal. Transmobilities enriches such perspectives by studying temporality and spatiality more explicitly. Empirically addressing the time dimension allows us to study not only short-term but also daily mobilities. This is especially important in border areas where migrants rely on daily cross-border mobilities such as Mexico–United States (Gauthier, 2012) and Chile–Bolivia (Jiménez, 2021). Further, considering scale makes it possible to understand that the repeated mobility of migrants between cross-border places can occur not only between distant places (e.g., Europe and Senegal) but also between close places in border zones (e.g. Colombia–Venezuela border). Likewise, these places can be located within the same country (local transmobilities) or within two or more countries (cross-border transmobilities). Finally, transmobilities of material and immaterial items by (former) migrants need not only to have a transnational character but may also take place within a country. In all cases, transnational networks are essential for transmobilities (Reynolds, 2010). The concept of transmobilities conveys the idea of ‘return’ not only as a singular and bi-directional movement between two geographical points (cf. Cassarino, 2004; Cavalcanti & Parella, 2013; Cortés & Oso, 2017; Ley and Kobayashi, 2005; Sinatti, 2011) but also as a set of multi-directional movements between different places and at different moments in life — an idea that I illustrate in Fig. 1, thus viewing return as a continuous rather than a completed movement across the globe. Return migration does not necessarily close the migration cycle (King & Kuschminder, 2022) but is always ‘a new beginning’ (Pauli, 2021, p.104).

Finally, how do we conceptually define ‘reintegration’? Successful reintegration is often measured solely in terms of economic achievement (Sinatti, 2011) without considering human rights and existential needs. Following the perspective of feminist geopolitics, I propose an integrated understanding of reintegration as the process through which a returnee attains the necessary conditions in which to use their capacities and resources in order to ensure that their fundamental, existential, and security needs are met. This conceptualisation thus integrates the principles of sustainable livelihoods, existential needs, and human-rights approaches. Furthermore, inspired by Chambers and Conway (1992), I define ‘sustainable livelihoods’ as income-earning activities that viably secure *fundamental human needs* such as food, shelter, water, energy, transport, medical care, and education in the long term. Building on the human-geographical concept of ‘sense of place’ (e.g. Tuan, 1977), I also address the *existential* dimensions of reintegration — the need to belong in a social space. Finally, in tune with the human-rights approach (UN, 1948), I also include *security needs* — feeling free from harm, persecution, and threat. This is particularly important for this study, as human-rights abuses are commonplace at the Colombian–Venezuelan border.

## Methods, Research Participants, and Sites

**Fig. 2:**
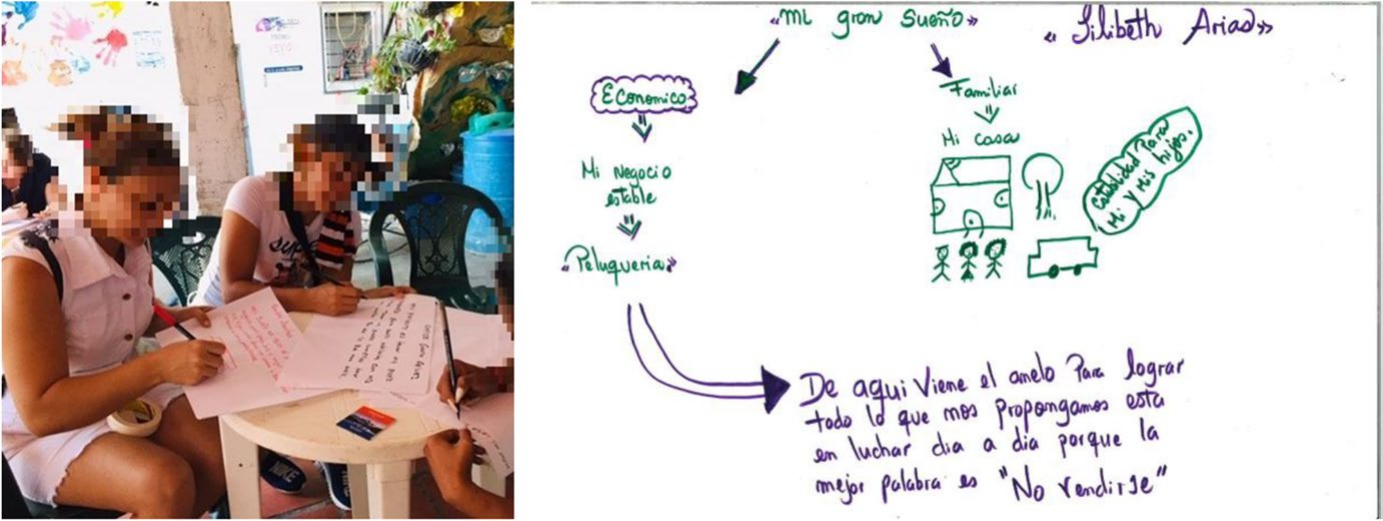
Mental maps, drawn by Colombian returnee women, of their reintegration aspirations during a participatory *Minga* workshop in 2019. *Translation*: ‘My big dream’. Economical: My stable hairdresser business. Family: My house as stability for my children and me. This is my yearning. To achieve everything we set out to achieve, we must fight day by day. The best words are ‘Don’t give up’ (Luciana Acosta^2^)

Qualitative fieldwork was conducted between 2019 and 2022. I interviewed 30 returnee entrepreneurs living between Colombia and Venezuela and conducted multi-sited ethnographic fieldwork with the support of a local NGO in Cúcuta. I used the method of life-narrative interviews (Dutta, 2016) to allow a comprehensive understanding of the migration and business trajectories of the studied entrepreneurs. I also draw on participatory *Minga* workshops (Fig. 2). This method, developed in my previous research (Riaño, 2016), creates ‘spaces of mutual learning’ where academics and non-academics jointly produce knowledge. While the *Minga* workshops created an environment of reciprocal learning, the method of mental maps (Jung, 2014) was applied during the workshops to visually capture the subjective perceptions of returnees regarding their entrepreneurial aspirations (Fig. 2). I used qualitative text analysis (Kuckartz, 2014) to identify key patterns in the collected data, thus combining deductive coding procedures (identifying a predetermined set of codes and then searching for excerpts that fit those codes) and inductive coding ones (developing new codes while the transcribed interviews are analysed) (Saldaña, 2021).

**Fig. 3:**
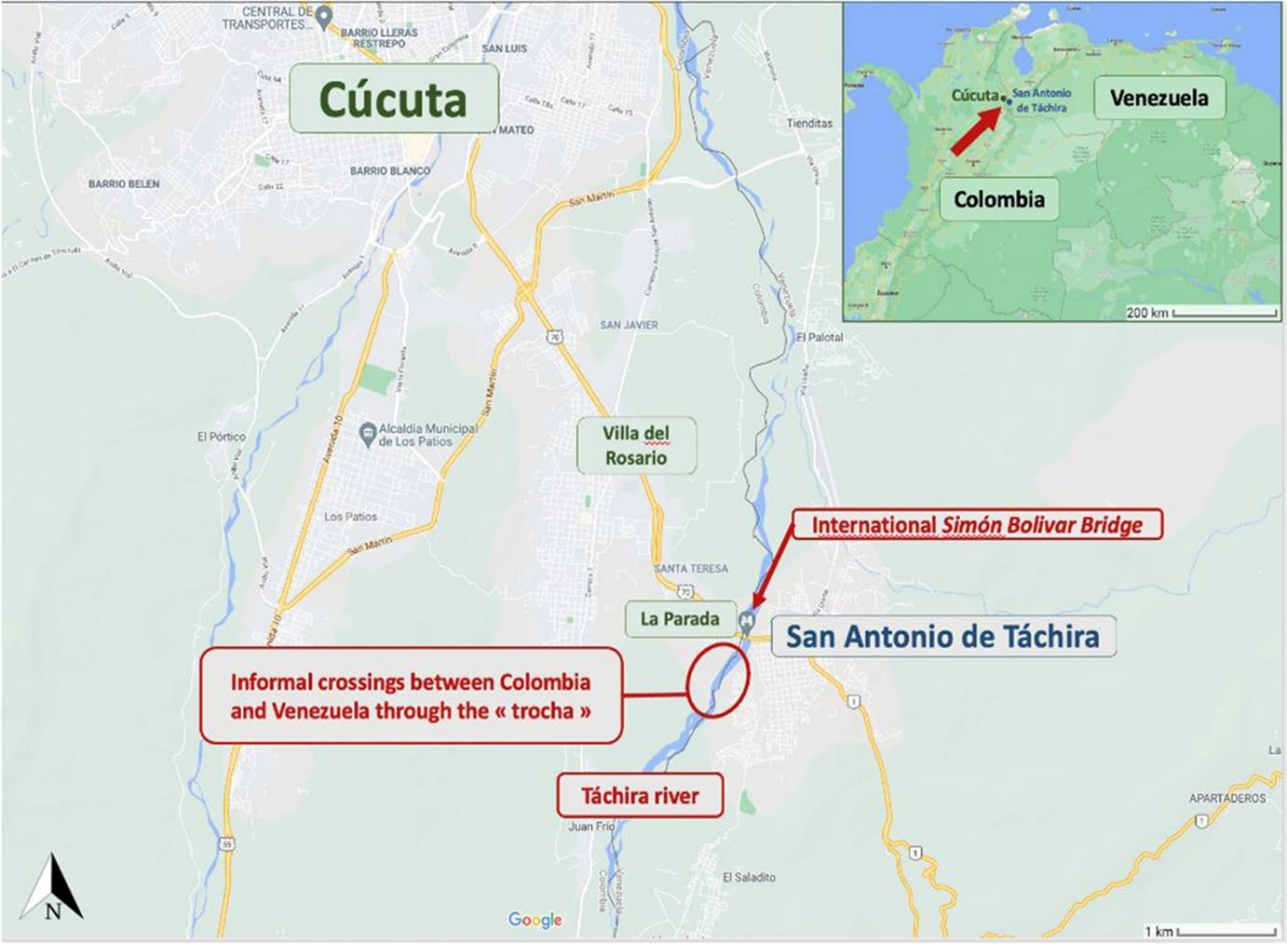
Fieldwork locations in the border area between Colombia (Cúcuta) and Venezuela (San Antonio de Táchira). *Source*: Figure composition and legend placement: Yvonne Riaño. Map data: Google ©2021, https://www.google.com/maps/search/cucuta+san+antonio/@7.8378349,-72.4708324,14.42z

Fieldwork was carried out at four sites: Cúcuta and La Parada (Colombia), San Antonio del Táchira (Venezuela), and the illegal border-crossing point between these countries, known locally as *la trocha* (Fig. 3). The relevant case studies were selected based on the principle of maximum-variation sampling (Glaser, 1992), which does not seek statistical representation but, rather, in-depth understanding of case studies that represent a variety of relevant variables including gender, age, residential location, and mobility experiences. The study sample consisted of 30 Colombians (25 women, 5 men). The majority (20) had basic education, were between 40 and 49 years old (13), had childcare responsibilities (29), migrated in the early 2000s to Venezuela primarily for reasons of territorial conflict (29), and were deported to Colombia in 2015 (29). They all set up micro-sized enterprises — mostly of an informal nature — such as food, textiles, handicrafts, leather goods, hairdressing, manicure services, transport, and social entrepreneurship aimed at helping deported Colombians and Venezuelan refugees. Table 1 sums up participants’ socio-demographic characteristics. The following sections detail the research results.

**Table 1:** Socio-demographic characteristics of returnees

Interviewed returnees	*n* = 30
Gender	
Female	25
Male	5
Age	
20–29	2
30–39	10
40–49	13
> 50	5
Child responsibilities	29
Highest educational level	
Primary school	20
Secondary school/vocational training	10
Primary reason for migration	
Territorial conflict	29
Work	1
Study	-
Family	-
Main business sector	
Food	10
Textiles/leather goods/handicrafts	8
Beauty services/hairdressing	6
Community building	4
Transport	2

## Geopolitics, Forced Mobilities, and Return

International and local geopolitics have shaped the forced mobilities of the returnees in this study. Most participants grew up in rural regions of Norte Santander, an area in Colombia’s north-east, bordering Venezuela. Given the lack of presence of the Colombian state in Norte Santander, illegal armed groups, including guerrilla and paramilitary groups, have disputed control of the territory for decades (as in other areas of the country). Fighting between guerrilla and paramilitary groups has forced many rural inhabitants to leave their homes and seek new livelihoods elsewhere. Most notably, illegal armed groups have threatened and killed many community leaders and human-rights defenders and conducted horrendous massacres of civilians (CNMH, 2015a; González et al., 2012). The most famous massacre took place in La Gabarra in 1999, a village near the border with Venezuela. In addition, guerrillas and paramilitaries also infiltrated low-income neighbourhoods of Cúcuta, Norte Santander’s capital city, causing terror among the civilian population. The dispute over the territory in Norte Santander left towns destroyed and displaced more than 34,000 people between 1997 and 2004 (CNMH, 2015b).

In the early 2000s, many Colombians, including all my research participants, emigrated to Venezuela, disillusioned by the lack of security in Norte Santander and by Colombia’s high cost of living. They were also attracted to the earning opportunities generated by soaring oil prices in Venezuela, as well as the social policies of Hugo Chávez’s government.

At first, Colombians experienced a very high standard of living in Venezuela — they could buy a house, set up a business and benefit from the low cost of living. This was possible because of the Chavez government’s *laissez-faire* approach to Colombians moving between Venezuela and Colombia for business, residence, tourism, and family visits. However, on 19 August 2015, the new president, Nicolás Maduro, ordered the closure of the Venezuelan border due to an alleged attack by Colombian paramilitary groups on members of Venezuela’s National Guard. This escalated into persecution against Colombians living in Venezuela. Accused of collaborating with paramilitary groups, some 2,000 Colombians were deported and more than 22,000 of their compatriots subsequently fled the country out of fear (Cosoy, 2015). The houses of many Colombians in San Antonio were subsequently destroyed by the National Guard and their inhabitants evicted and deported to Colombia. Many Colombians escaped to Cúcuta by crossing the *trocha* (the informal path through the bush) and the Táchira River (see Fig. 3). Some *deportados* stayed in Cúcuta, while others waited in camps until the situation calmed down before returning to San Antonio and repairing their homes (El Tiempo, 2020) (In what follows, all participants’ names are pseudonyms).

One of the female *deportadas*, Yolanda Buitrago, recounts her experience:

Since we were Colombians, they said that we were paramilitaries. And the houses of the Colombians that were marked with the letter ‘T’ were to be knocked down and the letter ‘D’ meant that it was in good condition and not to knock it down ... That was terrible, because you could see all the people crying over their houses, the collapsed houses, the refrigerators, everything under the roof. We got out what we could across the *trocha*.

Resulting from Norte Santander’s violent context, and the geopolitical conflicts between Colombia and Venezuela, most research participants have experienced human-rights abuse and forced displacement over the years. As shown in Table 2, more than 80% of the research participants were victims of internal displacement by guerrilla and paramilitary groups. Furthermore, all participants faced deportation either by physical or psychological force. Nearly half of the women were raped or suffered domestic violence. This trauma creates a difficult context for returnees’ reintegration. The results also show that returnees experience two different forms of forced return, namely violent deportation and fear of deportation, the characteristics of which are summarised in Table 3.

**Table 2:** Human rights abuses experienced by research participants

Human rights abuses	Number of individuals
Internal displacement	25 (out of a total of 30)
Deportation (by physical or psychological force)	29 (out of a total of 30)
Rape and domestic abuse	12 (out of 25 women)

**Table 3:** Experiences of forced return

	Women	Men	Total
Violent deportation	17	4	21
The Venezuelan government forces working Colombians to leave its territory through physical force by the police, damaging their houses, and destroying their residence permits			
Driven out by fear of deportation	8	1	9
Colombians leaving the Venezuelan territory out of fear that they will experience violent deportation			
Total	25	4	35

## Multiple Return Movements: Moored, Periodic and Daily Returns

My study identified three types of return movement or transmobility, which returnees practice for their livelihoods (Table 4). The first group, *moored return*, lives in Cúcuta (Colombia), and their daily mobilities revolve around this city. The second, *periodic return*, live in La Parada — a peripheral area of Cúcuta at the border with Venezuela — but they frequently return to the Venezuelan city of San Antonio del Táchira. The third, *daily return*, live in San Antonio del Táchira (Venezuela) but their daily transmobilities are towards Cúcuta (Colombia).

**Table 4:** Three types of return movements or transmobility among returnees

Type	Place of residence	Daily transmobilities
Moored return	Cúcuta, Colombia	Sleeping, working, health care, shopping, studying *within* Cúcuta
Periodic return	La Parada, a peripheral area of Cúcuta, at the border with San Antonio (Venezuela)	Sleeping, working, health care, shopping, studying in Cúcuta but *frequent mobility to San Antonio*
Daily returns	San Antonio (Venezuela) at the border with Colombia	Sleeping in San Antonio but *daily mobility to Cúcuta* to work, shop, study, access health care

Thus, research participants live transnational lives between Cúcuta and San Antonio del Táchira, which are connected by the 300-m-long Simón Bolívar Bridge across the Táchira River (see Fig. 3). Cúcuta and San Antonio are two distinct cities separated by a border which, for many of their inhabitants, is an imaginary line. Many people have relatives on both sides, including this project’s research participants — the product of cross-border mobilities over many years. Likewise, the Simón Bolívar Bridge is also the point of arrival for thousands of Venezuelans who, in recent years, have escaped their country’s economic and political crises to seek out better socio-economic opportunities in Colombia and elsewhere.

**Fig. 4:**
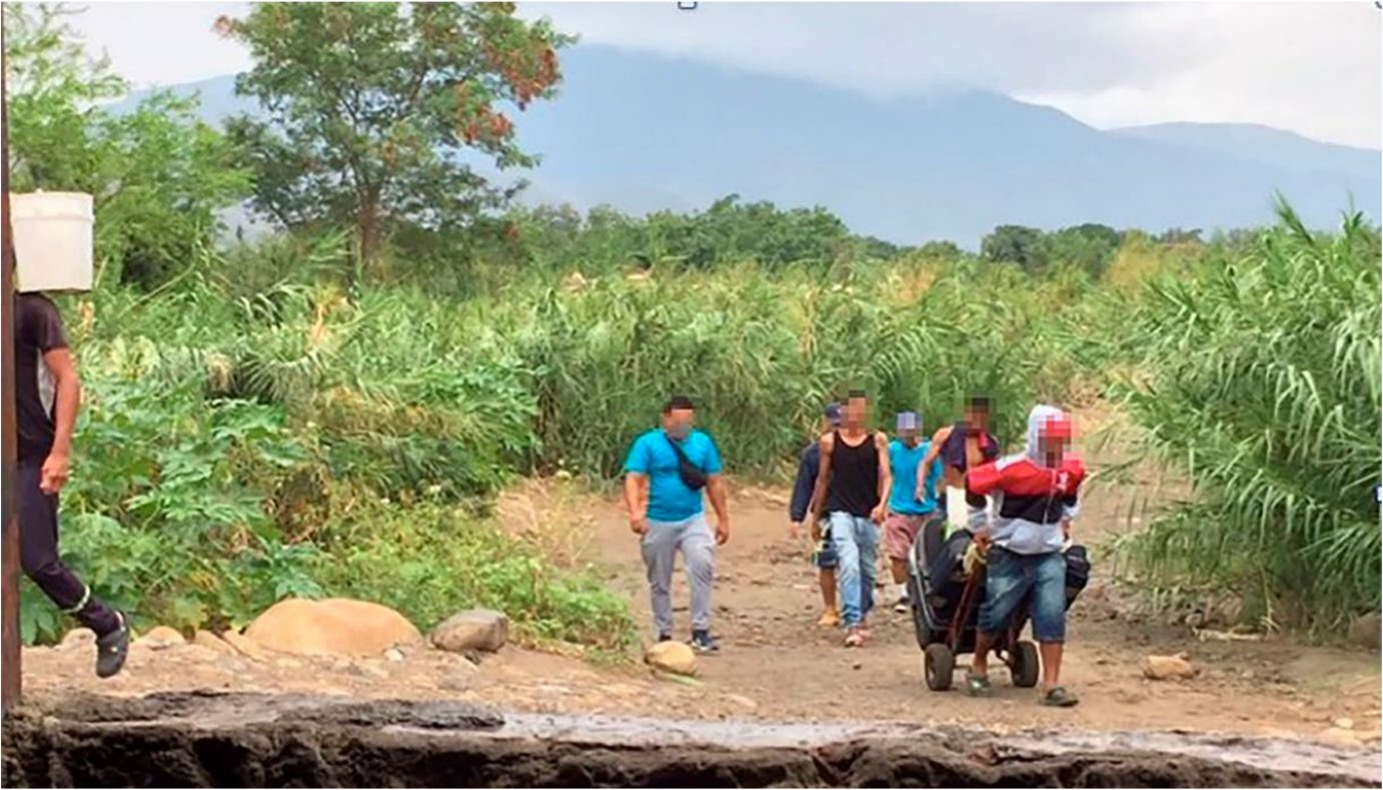
Informal transmobilities of people, goods, and capital across the *trocha* that connects Colombia and Venezuela. *Source*: Yvonne Riaño (2019)

Groceries, work, and educational opportunities are scarce in Venezuela but the ‘returnees’ who live there benefit from free housing, cheap electricity, water, gas, and oil. As the border is closed, they return, sometimes several times a day, to Colombia via the *trocha*, to work, conduct business, buy food, and benefit from access to Colombia’s superior educational institutions and health care (Fig. 4). However, movements across the *trocha* can be dangerous as the territory is controlled by illegal armed groups who threaten and extort money from informal crossers and impose their own form of criminal governance (Lessing, 2020).

The Venezuelan government unilaterally kept the official border closed between 2015 and the end of 2021. Paradoxically, the Colombian government kept it open but closed it for a brief period due to the COVID-19 pandemic. All this created much uncertainty and negative consequences for the businesses of the returnees studied here. In addition, although the border was opened in 2021, my most recent fieldwork in 2022 shows that many returnees prefer to use the *trocha* to cross the border because the corrupt Venezuelan police charge higher fees to let their products go through the international bridge than the guerrillas on the *trocha*.

## The Role of Entrepreneurship in Returnees’ Reintegration

This section examines the role of entrepreneurship in returnees’ reintegration, which is the core question of this paper. First, I explore what motivates returnees to become entrepreneurs. The interviews and the mental maps produced during the participatory workshops reveal that most returnees either already ran businesses in Venezuela and wanted to continue, believed it was the best way to support their families or had no other choice due to a lack of employment opportunities in Cúcuta. As participant Yoana Fuentes explains:

The saddest thing is to be healthy and not to have a job, because you go downtown to get a job and there aren’t any, because my husband and my son have gone and if you don’t have political connections, you can’t get a job. That is Cúcuta. If you have connections, you have work.

Cúcuta and its metropolitan area, to where most research participants return or rotate within, has had a relatively high average unemployment rate of 16 per cent over the past four years compared to Bogotá (11), Medellín (12), and Cali (12 per cent) (DANE, 2020). In addition, the influx of Venezuelans seeking job opportunities since 2015 has exacerbated the locational disadvantage for the interviewed returnees seeking employment. However, Cúcuta is where their family networks and potential accommodation are. Furthermore, the mental maps show that the interviewed returnees became entrepreneurs not simply for financial reasons but as a moral duty to help their families (see Fig. 2). These results suggest that we need to nuance the notion of ‘necessity entrepreneurship’ (Dencker et al., 2021). The motivations of the entrepreneurs studied do not result exclusively from negative circumstances such as unemployment but also from positive factors such as entrepreneurial experience and emotional factors including the desire to support their families.

To what extent can returnees successfully reintegrate as entrepreneurs? Earlier in the paper, I defined ‘successful reintegration’ as the on-going process by which a society provides the adequate framework conditions for a returnee to use their skills and resources to secure fundamental, existential, and security needs. The empirical results show that, despite remarkable resilience, commitment, creativeness, and personal resources in the form of skills and family support, most research participants struggle to reintegrate as entrepreneurs.

In terms of satisfying *fundamental needs*, which I defined earlier as ‘secure food, shelter, water, energy, transport, medical care, and education in such a manner that is viable long-term’, the empirical data reveal four types of situations, as seen in Table 5. All of these outcomes involve an element of uncertainty for the future, common across the sample of interviewees.

**Table 5:** Do returnees satisfy their fundamental needs?

(a) Barely satisfying fundamental needs
Returnees who could benefit from their migration trajectories and acquired skills but have no support from the government or international agencies and limited support from family
(b) Satisfying fundamental needs but uncertain future
Returnees who benefit from their migration trajectories, acquired skills, social contacts, and economic support from their families (daughter’s support, widow’s pension, housing in Colombia or Venezuela) but who have no international support and limited training from the Colombian SENA (National Learning Service) institution, owing to their limited education. The future of their entrepreneurship is uncertain
(c) Satisfying fundamental needs, potential for expansion but uncertain future
Returnees who benefit from their migration trajectories, acquired skills, social contacts, economic support from their families (daughter’s support, widow’s pension, housing in Colombia or Venezuela), some economic support from international agencies (loans, funding for activities), and SENA training but who cannot sell their properties in Venezuela. Their futures are uncertain because of a lack of state support in Colombia to obtain a loan
(d) Satisfying fundamental needs and expanding business but uncertain future
A returnee who benefits from his migration trajectory and acquired skills. He was able to sell his house in Venezuela because he lived far from the border, had social contacts and economic support from his family (was given bakery machinery by his mother, which he repaired), obtained loans from international agencies to buy raw materials, has professional training, and, as such, was able to profit from advanced SENA training. However, his business future is uncertain given the territorial conflict prevailing in Norte Santander

Regarding *existential needs*, support from family is essential to creating a sense of belonging. Without it, many returnees struggle for social belonging. This results from three types of situations. For a start, some who live in Cúcuta struggle to develop their businesses because it is difficult to overcome the trauma of deportation, as one research participant, Raquel Urriola, explains:

I believe that the first barrier that I have to overcome is to eliminate fear, by working on myself. I am trying to overcome fear, trying to gain security. I believe that what stops me, and even my husband, is the feeling of insecurity, which is like a feeling inside me that I can’t overcome.

Second, some who live in San Antonio del Táchira (Venezuela) but return daily to Colombia to satisfy their fundamental needs struggle to belong because they do not have homes in Colombia and fear being evicted from their Venezuelan homes, as has happened before. As their fear of crossing the dangerous *trocha* increases every day, living in Colombia is a strong desire, as research participant Rocío Calderón explains:

I now tell my partner that we should come to live [in Colombia] because I no longer want to be [in Venezuela] ... recently I was crossing the *trocha* with my girl when the shooting started and I got very scared for my girl and all that... Now the guerrillas are on the *trocha* and the other day the Colombian police got involved, so I’m afraid that a stray bullet will fall near us... Yesterday it was the Venezuelan police that got involved and there was shooting for a long time, so one does not have a life living [in Venezuela and crossing the *trocha* every day to come to Colombia]...But it is difficult [to settle in Colombia] because I am still trying to get a job but I cannot get a stable job because I have to look after my children ... and [in Colombia] it is difficult to live because [unlike in Venezuela] you have to pay rent, services. But I do want to come [to Colombia].

Third, many returnees experience so many challenges upon their return to Colombia that it is difficult for them to create a sense of social belonging. However, their spirit of resilience is remarkably strong and they continue to fight to achieve the desired reintegration, as participant Zoila Caicedo says:

I knew that in Colombia it would be difficult, because of the rent you have to pay. I used to have my own house; it is no longer the same to live now in a small room. But I know that all this is a struggle we have to go through, it is a mountain you need to tear down. You have to have faith.

Finally, I address the satisfaction of *security needs*, which was defined earlier as ‘feeling free from harm, persecution and threat’. It is clear that, in the case of returnees who cross the *trocha* every day, security needs are barely satisfied, as they are exposed to the risk of extortion and even death on a daily basis.

One day I was crossing the *trocha* with my children. There was a shootout. We ran off quickly, the porters protected us... yes, they showed us where to go... into the bush... but that is our life ... we live by the hand of God... (Yolanda Buitrago, 2020).

Those who live in La Parada, on the Colombian side of the border, currently experience extreme insecurity due to COVID-19 restrictions affecting cross-border mobilities. This has caused rampant poverty among Colombian and Venezuelan residents, as a recent interview with a local community leader, Adriana Cardona, shows. A similar situation is probably affecting all returnees living in the neighbourhoods of Cúcuta.

Here we do not leave the house anymore. There are a lot of thieves. They are stealing whatever they find. I emptied the kitchen yesterday. I left only the refrigerator and the stove, because they are taking things, pots, to sell them.

In sum, the empirical results show that the concept of successful reintegration does not apply to the majority of the interviewees since they are not able to achieve their fundamental, existential, and security needs sufficiently. In line with Riaño (2013) and Mensah (2016), we can conclude that Colombian returnees experience high levels of dependency on family networks and (some) on international support and undertake dangerous mobilities in order to survive, given the weak governance and the absence of effective national reintegration policies. Why does this happen? What are the main challenges that returnees face?

To begin with, the geopolitical context in which the returnees live is highly unfavourable. The struggle for territory between illegal armed groups in Colombia, the inability of the Colombian state to control this conflict and the uncertainty created by the arbitrary closures of the international border by the Venezuelan government create a highly vulnerable environment for returnee entrepreneurs. Additionally, the trauma of deportation and the consequent loss of livelihood plus the costs of resettlement negatively impact the development of returnees’ businesses. Furthermore, the lack of effective economic support from the Colombian government is a major challenge, as one interviewee, Valeria Barrera, explains:

Here in Colombia, even though I’m Colombian, I don’t have any help. The government does not help at all ... neither as an internally displaced person, nor as a returnee, nor as a victim of sexual violence, nor as a single mother.

However, the problem is not a lack of Colombian institutions or laws to protect the displaced, deportees, and returnees. The goal of the Unit for the Care and Reparation of the Victims of the Armed Conflict (UAV) is ‘to lead actions of the State and society to fully attend and repair the victims, to contribute to social inclusion and peace’. Unfortunately, the interviewees were unable to obtain support from this institution due to extreme bureaucracy. In addition, the 2012 Return Law (Law 1565) is intended to qualify returnee entrepreneurs for financial and logistical support from the *Fondo Emprender SENA*.^2^ In practice, however, this programme is designed for people with higher education who are developing innovative technologies. This reflects how return policies are primarily designed for highly skilled migrants with available capital. Furthermore, financial institutions in Colombia require guarantees in the form of property, a co-signer, and proof that the business exists and is performing well. None of the returnees whom I interviewed meet these conditions, making such policies clearly inadequate. Having lived abroad for many years, they cannot prove that their businesses have already started and are promising and they have no collateral or financial backing with which to support their loans. Furthermore, their economic capital acquired in Venezuela was devalued by hyperinflation. For deportees, it is impossible to sell their property in Venezuela in order to invest the profit in their businesses.

Many returnees also decried a lack of information and difficulties accessing the Internet. Most do not have an Internet connection at home and therefore must pay for it per usage outside the home, which is impossible for many. The only seemingly effective Colombian institution is the SENA (National Learning Service), which trained several of the interviewees in business management and basic manufacturing techniques. However, many interviewees lack secondary-level training, a SENA requirement for many of the apprenticeship programmes. The interviewees do not have the time or financial resources to complete a secondary-level education since they must work to subsist.

Finally, multiple burdens are placed on female entrepreneurs, who must reconcile childcare and paid work alongside the traumas caused by internal displacement, deportation, daily uncertainty, rape and domestic abuse. Lastly, COVID-19 makes the survival of the research participants’ businesses particularly challenging both because they were not allowed to go out to sell during the pandemic lockdown and because their informal businesses often take place on the streets, which was prohibited in 2020 and part of 2021.

## The Role of Transmobilities for Returnee Entrepreneurs

To what extent do returnees use the strategy of transmobilities in their entrepreneurship? Nearly half of the studied group cross national borders every day. This means, firstly, that they use their geographical proximity to the border, their spatial knowledge, circulatory expertise, and transnational trade contacts to mobilise themselves or others across the *trocha* to sell goods such as food and handicrafts or to provide transport, cleaning, hairdressing, and manicure services. In this case, using transmobilities as a strategy for entrepreneurship is not an advantage for expansion but, rather, a strategy to survive.

Although transmobility offers potential advantages for the former entrepreneurs, others are less dependent on subjecting themselves — or others — to the dangers of crossing the *trocha* everyday as they rely on the support of international organisations for their entrepreneurial activities. For example, some obtain economic capital and training from agencies such as the Scalabrini International Network (a community of missionaries supporting migrants, refugees, and displaced people) and the German Cooperation for International Development (GIZ). Returnees who benefit from this international support generally have better prospects for their economic and social entrepreneurship ventures but rely on foreign aid.

In short, transnational mobilities and transnational networks, whether in the form of international agencies or informal contacts, are essential for the entrepreneurial success of all returnees. This raises the question of dependency on foreign aid and on informal networks that might become vulnerable over time. Any sustainable development policy seeks to avoid dependence on foreign support in the long term.

I now present two brief biographies of Enrique Villarín and Sandy Barroso, which illustrate how Colombian returnees from Venezuela use the strategy of transmobility of goods, capital, and people to develop their businesses. They also illustrate that the kinds of opportunity and constraint that returnees face are underpinned by the geopolitical situation that characterises the border area between Colombia and Venezuela.

### Enrique Villarín

Enrique Villarín is a 38-year-old Colombian. He grew up in an area controlled by the FARC guerrillas. In 1992, his family was forcibly displaced after armed right-wing paramilitaries accused them of cooperating with the FARC. They changed residence several times over the years, fleeing a massacre at one point. The family escaped to Cúcuta (Colombia). There, they learned to sew jeans and t-shirts and commuted daily to San Antonio del Táchira (Venezuela) to work.

In 2007, they migrated to Venezuela, set up a business in San Antonio del Táchira to produce clothing to sell in Colombia and Venezuela and came by a property in Venezuela near the border. When Venezuelan President Maduro closed the border in 2015, Enrique and his family escaped to Cúcuta but returned to San Antonio six months later. In 2020, despite still having equipment, Enrique’s family was unable to restart their manufacturing business because they lacked the capital to buy raw materials. Enrique was denied credit in Colombia because he had no financial guarantor.

In the absence of support from the Colombian government and with the border closed, Enrique put his business on hold. He temporarily used his body, his transnational circulatory experience, and his entrepreneurial and family networks to become a *maletero* or freelance porter who risks his life illegally carrying goods across the *trocha*. Enrique says:

My dream is to have a clothing factory to be able to support my family and also help those most in need and provide my knowledge to those who need it, so that they can get ahead and not depend on anyone.

Enrique’s economic activities are part of a complex transnational informal economy at the Colombia–Venezuela border.

Enrique’s account illustrates his skills, resources, and challenges: circulatory and professional knowledge, manufacturing equipment, property in Venezuela, local and transnational business networks but no investment capital or government support. Transnational networks and transnational mobilities are key to his survival. Enrique’s case exemplifies a potential entrepreneur who cannot progress due to a lack of development capital. Thus, he sells his personal mobility, which allows him to transform spatial mobility into economic resources but at great risk to his life. His transmobilities are both enabled and hampered by the geopolitics of border and conflict management.

### Sandy Barroso

Sandy Barroso is a 46-year-old from Colombia’s coffee growing region. She left home at 15, married, and had three sons. In 1993, she and her children emigrated to Venezuela to escape her violent husband and the threat of illegal armed groups. There, they first worked as travelling traders selling hand-made earrings at fairs in various cities, settling in Guanare (430 km from Cúcuta) near her mother and sister.

Sandy obtained a food-stall licence to sell coffee and baked goods. She also sold Colombian products at weekend fairs. She returned monthly to Cúcuta to visit her new husband and buy new merchandise. Initially, she managed to save money and invest in her business. However, as the Venezuelan Bolivar plummeted in value and the cost of living rose, Sandy struggled to survive. In 2015, she witnessed the Venezuelan government deporting Colombians. In fear, she and her mother left everything behind — including a house — and returned to Colombia.

They travelled to San Antonio and reached the *trocha*, where Venezuelan guards threatened to take them prisoner. They paid *maleteros* guides to transport their few goods to Colombia along the *trocha*. Arriving in Cúcuta, Sandy stayed at her husband’s home and set up a bazaar. The Jesuit Refugee Service, an international organisation assisting victims of forced displacement, helped her with food and business training. She now sells her products to her neighbours and to clients in Venezuela, which she delivers to San Antonio for transportation to Guanare.

On other occasions, one of Sandy’s Guanare customers comes to Cúcuta to collect her handicrafts, which he later sells at his street stall in Venezuela. Her sales are intermittent but private and public celebrations offer good opportunities to sell party decorations. Her dream is ‘to have a large bazaar with a good array of products’. Unfortunately, Sandy lacks the capital to expand her business — her loan requests to Colombian institutions have been unsuccessful. Sandy’s case illustrates how informal street-trading networks develop internationally and the difficulties which traders face.

Her story also illustrates the kind of resources that returnees possess: circulatory and professional knowledge, her husband’s house in Cucuta, and useful support from NGOs for learning handicrafts. She demonstrates the use of resources to survive without risking her life but on a very small scale and with uncertain prospects. Sandy’s case also illustrates how transnational mobilities and transnational networks are an asset for her business but not indispensable. However, if relations between Venezuela and Colombia were to improve, this could be a great business asset.

What do these two biographies tell us? They illustrate how many returnees from Venezuela, as well as inhabitants of Cúcuta and San Antonio, use their everyday agency to recreate international borders. Despite Maduro’s imposition of a hard border between Colombia and Venezuela, residents of the area use formal and informal transmobilities to maintain the permanent circulation of people, goods, and capital between Cúcuta and San Antonio, thus creating strong socio-economic and emotional connections between the two cities. These results illustrate my theoretical argument that ‘return’ needs to be understood as a continuum of *transmobilities* framed by the strategies that individuals activate within a transnational space.

## Conclusions

This paper addresses some open questions in the literature on migrant return — namely, the extent to which returnees can successfully reintegrate with their entrepreneurial ventures. To grasp this complex question, I use ethnographic field work, biographical interviews, mental maps, and participatory *Minga* workshops (Riaño, 2016) with 30 Colombian migrants returning from Venezuela. Further, I use a *feminist geopolitical* perspective of study thus emphasising the role of territorial struggles where returnees are attempting to reintegrate and agency. I bring together the ideas of transnationalism and mobilities to offer the concept of *transmobilitie*, which examines how (former) migrants mobilise goods, capital, and other people across national borders as a critical strategy to reintegrate. This conceptual approach contributes to advancing our understanding of return movements and reintegration through entrepreneurship.

The return movements of the studied persons emerged as heterogeneous. Returnees practice three types of transmobility: (a) *moored return* (residing in Cúcuta, Colombia, with daily transmobilities within this city); (b) *periodic return* (residing near the border, with frequent transmobilities between Colombia and Venezuela); and (c) *daily returns* (residing in Venezuela with daily transmobilities to Colombia).

Furthermore, the results reveal a continuum of situations ranging from forced to chosen mobilities, which are contingent upon the unfolding geopolitics of migration and return. Two forms of *forced mobility* exist, which I label ‘violent deportation’ and ‘fear of deportation’. In contrast, two forms of *chosen mobility* are identified: ‘voluntary but experiencing some constraints’ and ‘fully voluntary’. These differences arise because some returnees have agency over their spatial mobilities (or immobilities) while others have mobilities imposed upon them. Using Massey’s term (1994), we can thus speak of different ‘power geometries’.

Regarding the issue of the extent to which returnee entrepreneurs can successfully reintegrate — considering the kinds of constraint and opportunity they face — the results show that, despite remarkable resilience, commitment, creativity, and personal resources in the form of skills and family support, most returnees face ongoing precariousness. They struggle to satisfy their fundamental, existential, and security needs in the face of a number of challenges. For a start, the geopolitical context they are situated in is characterised by territorial struggles between illegal armed groups, the absence of the Colombian state, and the uncertainty created by ongoing closures of the international border. This creates a highly vulnerable environment for entrepreneurs. Besides, the trauma of deportation and the consequent loss of their livelihoods negatively impact the development of their businesses. Furthermore, a lack of effective economic support from the Colombian government is a major challenge. Existing credit-support programmes are conceived for those with professional or university training and businesses with innovative technologies; they are not suitable for returnees who, having lived abroad for years, cannot meet the criteria for a loan. Additionally, the returnees in my study are not supported by banks because their personal savings were devalued due to hyperinflation in Venezuela. Moreover, a particular burden is placed on women, who juggle childcare and paid work while dealing with the traumas of one or (usually) more of the following — internal displacement, deportation, daily uncertainty, and sexual and domestic abuse. Return migration is a gendered process (King & Lulle, 2022) and the effects of gender relations and power dynamics can be particularly brutal on women returning to countries with territorial conflicts.

The argument that the economic capital brought in by migrants from abroad can be an important entrepreneurial resource upon return needs to be reassessed, especially in the context of disruptive geopolitics and border regimes. Not all countries to which migrants return offer adequate conditions in which to use the economic capital gained during migration. Moreover, not all migrants return from rich and stable countries in the Global North; many return from unstable countries such as Venezuela. The returnees whom I studied may not be highly skilled but they have great potential to create small businesses that stimulate local development. It is thus important to move beyond the primarily optimistic nature of debates on return migration and development and, instead, make a differentiated analysis of who is able to mobilise international resources and who is not, where and why and to devise adequate policies for returnees to employ international resources. More research and targeted policies are necessary to make this happen.

*Transmobilities* are key to the reintegration of returnee entrepreneurs. Moreover, informal transmobilities of people, goods, and capital across the Colombian–Venezuelan border are part of returnees’ struggles between territorial conflict and securing a livelihood. Dominant forms of territoriality are thus contested by the informal cross-border mobilities of Colombian returnees, which illustrates what Keith and Pile (1997) call ‘geographies of resistance’. However, resistance strategies need to be critically examined as they also encompass dangerous mobilities and the violation of human rights, as this paper has shown. Furthermore, dependency on transnational mobilities and international agencies is far from ideal because this creates other types of spatial, political, and social dependencies, the long-term sustainability of which is not guaranteed. Any sustainable development policy seeks to avoid dependence on foreign aid in the long term. Support from regional and national governments is thus essential to ensuring long-term sustainable reintegration and the protection of returnees’ human rights.

Finally, using a feminist geopolitical perspective proved useful in understanding that *international and local geopolitics* shape the transmobilities of returnees as well as their reintegration processes. The results reveal that territorial conflicts are not just between nations. Returnees’ agency plays a key role in the recreation of international borders through the informal transmobility strategies they use to cope with immobilities imposed by state regulations and armed forces. By questioning security at the scale of the individual body, as opposed to that of the nation state, our understanding of security is fundamentally transformed (cf. Hyndman, 2008) as well as our comprehension of the ways that fear and risk are actually experienced, resisted and contested by differently situated returnee populations. Security risks trickle down to the most intimate spheres of migrants’ lives. Furthermore, non-governmental and non-profit organisations are essential to the geopolitics of displacement since they assist migrant returnees who are victims of forced displacement. Finally, there is no accountability for the harsh politics and practices of return/deportation in South–South contexts. The condition of deportability has been seen as the product of immigration laws that construct a certain category of immigrants as ‘illegal’ (de Genova, 2002). However, in this case study, deportation does not even respond to legal criteria but to the simple abuse of human rights. This needs to be further investigated and seriously addressed by the international community.

## Footnotes

1 The term ‘transmobilities’ has so far been used in urban studies to examine transgender individuals’ experiences of harassment on public transport (Lubitow et al., 2017).

2 The programme *Colombia Nos Une* of the Directorate of Migration and Consular Affairs of the Foreign Ministry, in coordination with the International Organization for Migration (IOM), leads this initiative.
